# In Vitro Assessment of the Expression and T Cell Immunogenicity of the Tumor-Associated Antigens BORIS, MUC1, hTERT, MAGE-A3 and Sp17 in Uterine Cancer

**DOI:** 10.3390/ijms17091525

**Published:** 2016-09-09

**Authors:** Anke Vanderstraeten, Sandra Tuyaerts, Tina Everaert, Rieta Van Bree, Godelieve Verbist, Cathérine Luyten, Frederic Amant

**Affiliations:** 1 Department of Oncology, Gynecologic Oncology, Campus Gasthuisberg, Sandra Tuyaerts, KU Leuven—University of Leuven, Herestraat 49 Box 818, B-3000 Leuven, Belgium; anke.vanderstraeten@gmail.com (A.V.); tina.everaert@uzleuven.be (T.E.); rita.vanbree@kuleuven.be (R.V.B.); katrien.luyten@kuleuven.be (C.L.); frederic.amant@uzleuven.be (F.A.); 2 Department of Gynecology and Obstetrics, Division Gynecologic Oncology, University Hospitals Leuven, B-3000 Leuven, Belgium; godelieve.verbist@kuleuven.be

**Keywords:** tumor-associated antigens, expression, immunogenicity, endometrial carcinoma, uterine sarcoma

## Abstract

Background: While immunotherapy moved to the forefront of treatment of various cancers, it remains underexplored for uterine cancer. This might be due to the small patient population with advanced endometrial carcinoma and uterine sarcoma. Data about immunotherapeutic targets are scarce in endometrial carcinoma and lacking in uterine sarcoma. Methods: Expression of five tumor-associated antigens (TAA) (BORIS, MUC1, hTERT, MAGE-A3 and Sp17) was validated in uterine tumor samples by immunohistochemistry (IHC) and/or quantitative reverse-transcriptase polymerase chain reaction (qRT-PCR). TAA immunogenicity was analyzed by determining spontaneous T cell responses towards overlapping peptide pools covering the whole TAA in patient blood. Results: At mRNA level, *MAGE-A3* and *Sp17* were overexpressed in a minority of patients and *BORIS* was moderately overexpressed (26% in endometrial carcinoma and 62% in uterine sarcoma). *hTERT* was overexpressed in the vast majority of tumors. On protein level, MUC1 was upregulated in primary, recurrent and metastatic EMCAR and in metastatic US tumors. hTERT protein was highly expressed in both normal and malignant tissue. Spontaneous TAA-specific T cell responses were detected in a minority of patients, except for hTERT to which T cell responses occurred more frequently. Conclusions: These data point to MUC1 and hTERT as most suitable targets based on expression levels and T cell immunogenicity for use in immunotherapeutic regimens.

## 1. Introduction

Cancer immunotherapy, based on the recognition of the so-called tumor-associated antigens (TAA), is a valid therapeutic option for many types of cancer. Numerous different TAA have already been described to date. In 2009, Cheever et al. ranked a list of known TAA according to a whole array of characteristics, such as tissue expression, presentation in major histocompatibility complex (MHC) context, and immunogenicity [1]. Several TAA have already been described in various tumor types and tested in (pre)clinical immunotherapeutic settings with varying success [2]. Most data have been gathered in among others melanoma [3,4,5,6], glioblastoma [7,8], renal cell carcinoma (RCC) [9], prostate cancer [8] and hematological malignancies [10].

Tumor immunology and immunotherapy are, however, largely underexplored in uterine cancer. Although some of these known antigens have already been described in uterine cancer, only few studies were done to verify their potential use in immunotherapeutic strategies. Uterine tumors have been shown to be immunogenic, which is exemplified by the presence of tumor-infiltrating lymphocytes and macrophages [11,12]. Some immunotherapy studies have already been performed in uterine tumors using WT1 as target antigen [5,13,14]. Other (pre)clinical studies in uterine tumors based on the targeting of antigens have mainly focused on the use of whole tumor lysate [15,16]. The current research is focused on the validation of some of the described TAA in uterine tumors. Specifically, we have analyzed the following antigens: brother of the regulator of imprinted sites (BORIS), mucin-1 (MUC1), human telomerase reverse transcriptase (hTERT), sperm protein 17 (Sp17) and melanoma-associated antigen A3 (MAGE-A3). BORIS, Sp17 and MAGE-A3 are members of the cancer-testis antigen family. In normal tissues, their expression is restricted to male germ cells, fetal ovary, placenta and immature gametes [17], while they are expressed in several types of cancer, among which breast cancer [18,19], making them attractive targets for immunotherapeutic purposes. hTERT is considered as a universal TAA. This antigen plays an essential role in the survival of tumor cells, which makes it particularly attractive. It is absent in adult tissues, with the exception of proliferating tissues, while being up-regulated in dividing tumor cells, which fortifies its suitability as target TAA [20,21]. MUC1 is an epithelial antigen, constitutively expressed on normal epithelium in its wild-type form. The TAA MUC1, however, discriminates itself from the wild-type form by a hypoglycosylation pattern, exposing new epitopes, which can attract antigen-specific T cells [22]. These antigens were arbitrarily selected based on an analysis of the available data of these antigens concerning: (a) their presence in uterine tumors; (b) their ranking in the prioritization list described above; (c) available data on their application in immunotherapeutic regimens in other tumor types; and (d) their specific expression/function in tumorigenesis.

Besides these shared TAA, recently, patient-specific mutations have attracted attention as tumor-specific neoantigens that are recognized by specific T cells and to which no immune tolerance is present [23,24,25]. Hence, these neoantigens most probably constitute the ideal targets of immunotherapy. The presence of neoantigens has been described in endometrial cancer, predominantly in polymerase E (POLE)-mutated and microsatellite instable (MSI) tumors, which is consistent with the high mutation burden in these tumors [26]. However, the identification of neoantigens is a cumbersome and time-consuming process, which makes it difficult to implement in immunotherapeutic approaches. To our knowledge, neoantigens have not been described in uterine sarcoma, which could be explained by the fact that these tumors contain less point mutations but contain rather complex chromosomal rearrangements. One type of these rearrangements, however, chromosomal translocations, can give rise to the formation of unique new fusion proteins, which could be recognized by the immune system [27,28,29]. However, this phenomenon has not been described yet in translocation containing uterine sarcomas [30].

We currently aim to validate the presence of the above-described shared TAA in endometrial carcinomas and evaluate their expression in uterine sarcoma, which has, to our knowledge, not been described before. In addition, we evaluate their immunogenicity by analyzing the naturally-occurring in vivo TAA-specific T cell responses. The combined data will give a view on the applicability of the currently investigated shared TAA for immunotherapy in uterine cancer patients.

## 2. Results

### 2.1. TAA Expression in Normal Human Tissues and Tumor Cell Lines at mRNA Level

Prior to testing primary patient material, mRNA expression levels of the different TAA were assessed on a panel of normal human tissues. Of the currently selected antigens, PCR analysis was performed for all antigens, except for MUC1. The difference between the wild-type and tumor antigen MUC1 is a difference in glycosylation pattern. This is a post-translational modification and thus cannot be detected at the level of RNA. As expected, the cancer-testis antigens *BORIS*, *MAGE-A3* and *Sp17* were expressed at highest levels in testis, but for *BORIS* and *Sp17* a fairly high level of background expression could also be observed in other tissues. *MAGE-A3* expression was more restricted to testis (Table S1). With regard to the universal TAA *hTERT*, the highest expression levels were found, as expected, in tissues with a lot of proliferative cells, such as spleen, testis, and thymus (Table S1).

TAA expression was also assessed on a small selection of commercially available gynecological tumor cell lines. All TAA were expressed at medium to high levels in these cell lines, except for *MAGE-A3*, which showed lower expression levels (Table S1).

### 2.2. TAA Expression in Uterine Cancer at mRNA Level

**Figure 1:**
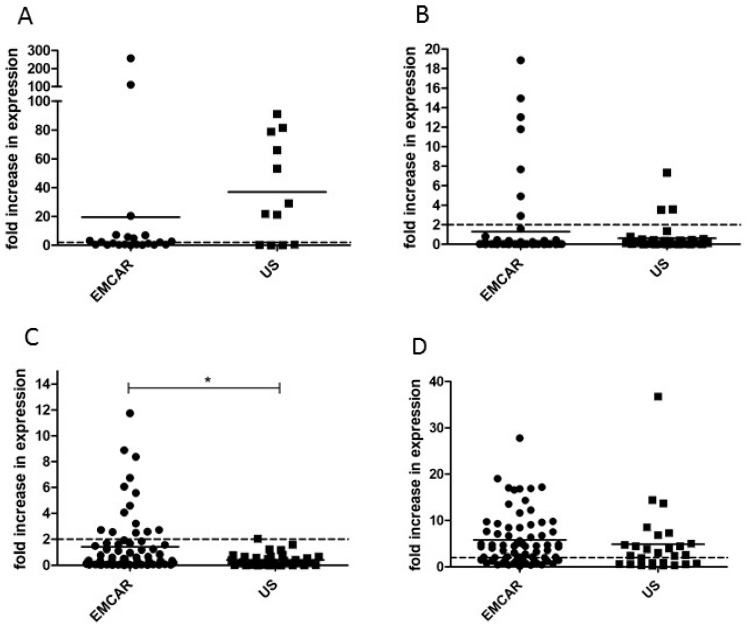
mRNA expression of TAA in uterine tumors: (**A**) *BORIS* expression; (**B**) *MAGE-A3*; (**C**) *Sp17*; and (**D**) *hTERT*. Results are expressed as fold change compared to the mean normalized expression of normal endometrium. Overexpression is defined as two-fold increase compared to normal endometrium (dashed line). TAA: tumor-associated antigen; EMCAR: endometrial carcinoma; US: uterine sarcoma; BORIS: brother of the regulator of imprinted sites; MAGE-A3: melanoma-associated antigen A3; Sp17: sperm protein 17; hTERT: human telomerase reverse transcriptase; * *p* < 0.05.

When looking at the mRNA expression levels of *BORIS* in our sample collection, we noted that *BORIS* was detectable in 60% of healthy controls, 50% of endometrial carcinomas and 92% of uterine sarcomas. Furthermore, we observed *BORIS* overexpression (calculated as >2-fold increase compared with the mean expression in healthy controls) in 26% of endometrial carcinomas and 62% of uterine sarcomas (Table 1). The expression seems slightly, yet not significantly, increased in uterine sarcoma (US) compared with endometrial carcinoma (EMCAR; Figure 1A). Due to a fairly small amount of samples tested for this antigen, no analysis concerning clinicopathological features was performed. All clinicopathological features of the samples that were currently analyzed are summarized in Table S2.

*MAGE-A3* could be detected in almost all samples (100% of controls, 90% EMCAR and 91% US) and was overexpressed at mRNA level in 10% of EMCAR samples and 9% of US samples (Table 1, Figure 1B). No differences in *MAGE-A3* expression were observed among the different FIGO stages of EMCAR, nor between the different histological subtypes or grade of differentiation of EMCAR or histological subtypes of US (data not shown).

*Sp17* transcripts could be detected in all samples. Overexpression of *Sp17* mRNA was found in 20% of samples of the EMCAR population and in 3% of uterine sarcomas (Table 1). It was significantly up-regulated in EMCAR compared with US (*p* = 0.0273, Figure 1C). In addition, a significant down-regulation in type II EMCAR compared with type I EMCAR was found (*p* = 0.0206), indicating potential down-regulation of *Sp17* with increased aggressiveness of tumors (data not shown).

We noted that *hTERT* was detectable in 33% of control samples, 100% of EMCAR and 21% of US samples. *hTERT* mRNA was overexpressed in 71% and 48% of EMCAR and US, respectively (Table 1, Figure 1D). When comparing *hTERT* expression in EMCAR according to FIGO stage, *hTERT* was up-regulated in stages II (*p* < 0.01), III (*p* < 0.01) and IV (*p* < 0.05) compared to stage I (data not shown).

### 2.3. TAA Expression in Normal Human Tissues at Protein Level

Protein analysis was done only for hTERT and MUC1. For the other antigens, no optimal specific antibody was available. For MAGE-A3 there is no commercially available antibody that is specific for the detection of MAGE-A3; TAA expression in normal tissues was evaluated using a TMA containing 22 different normal tissues. We found that 42.9% expressed MUC1. As expected, expression was predominantly found in epithelial cells. In addition, 57.6% of tissues expressed hTERT. The tissues that express each of the investigated antigens and the respective scores are summarized in Table S3.

### 2.4. TAA Expression in Uterine Cancer at Protein Level

**Figure 2:**
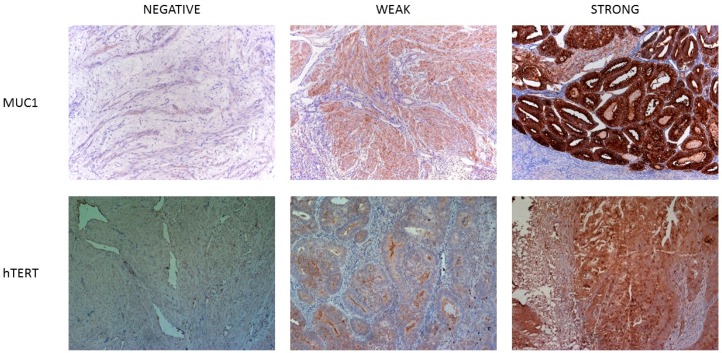
Immunohistochemical staining of MUC1 and hTERT in uterine cancer tissues. In this picture, we show a representative picture of a negative, weak and strong staining pattern for both antigens. Pictures were taken at a 10× magnification.

For protein expression analysis of MUC1 and hTERT a broad spectrum of paraffin-embedded tissue samples were analyzed. A representative picture of a negative, weak and intense staining pattern of MUC1 and hTERT is shown in Figure 2. All clinicopathological characteristics are summarized in Table S4. In addition, in order to obtain an idea on the expression of a certain antigen in the tumor as a whole, specimens taken at different locations in the tumor, to a maximum of three different sites, were analyzed if possible. The total number of tumors that were analyzed for each of the antigens is summarized in Table 2.

**Figure 3:**
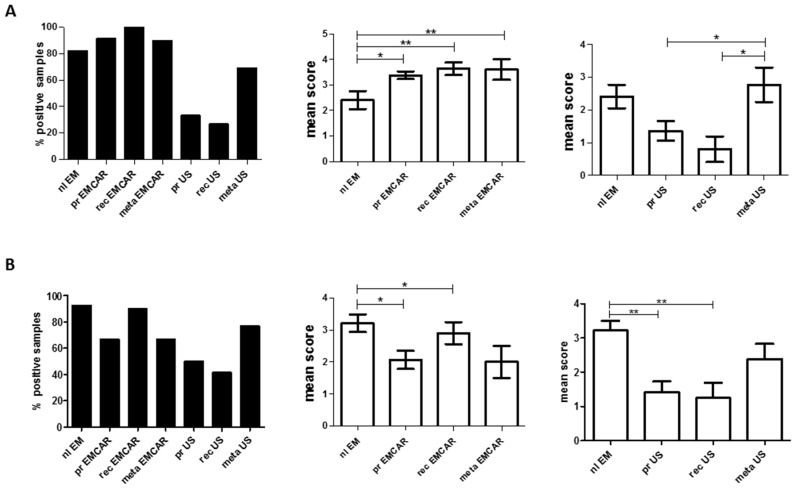
Expression levels of TAA by IHC: (**Left**) overall percentage of positive biopsies; **(Middle**) TAA expression levels in normal endometrium and EMCAR samples; and (**Right**) TAA expression levels in normal endometrium and US samples. (**A**) MUC1 expression; and (**B**) hTERT levels. nl EM: normal endometrium; pr EMCAR: primary endometrial carcinoma; rec EMCAR: recurrent endometrial carcinoma; meta EMCAR: metastasis endometrial carcinoma; pr US: primary uterine sarcoma; rec US: recurrent uterine sarcoma; meta US: metastasis uterine sarcoma. * *p* < 0.05; ** *p* < 0.01.

Microscopic analysis of the tissue samples showed that for MUC1, 96.6% of the pooled EMCAR population expressed the antigen. In comparison with normal endometrium, primary tumors (*p* < 0.05) as well as recurrent tumors (*p* < 0.01) and metastatic lesions (*p* < 0.01) show a significant up-regulation (Figure 3A) based on slide scoring. In addition, 43% of the US samples was also positive for this tumor antigen. For this type of tumors, the metastatic lesions show a significant up-regulation of MUC1 in comparison to the primary and recurrent tumors (*p* < 0.05; Figure 3A). This difference is most likely due to the high total amount of carcinosarcomas and endometrial stromal sarcomas in the total population of metastases (9/13). However, in the recurrent tumor population 8/13 tumors have either histological subtype, indicating that MUC1 is indeed expressed at the highest level in metastatic lesions. For a small number of patients, we had paired biopsies of the primary tumor and/or metastatic tumor and/or recurrent tumor in our data collection for which the scores are shown in Table S5. In addition, these data point to an upregulation of MUC1 in metastatic and recurrent tumors, although this analysis has to be interpreted with caution due to the limited number of patients.

The protein data for hTERT showed positivity in 75.6% of EMCAR samples and in 59.3% of US samples. Remarkably, the antigen was significantly up-regulated in normal endometria in comparison to primary EMCAR and metastatic EMCAR locations (*p* < 0.05) as well as primary and recurrent US (*p* < 0.01, Figure 3B). In addition, for hTERT, we show in Table S5 the scores of the IHC staining in a small number of patients with paired biopsies of primary and/or metastatic and/or recurrent tumors. However, since the hTERT stained section of the primary tumors of two of these eight patients were impossible to score due to extensive tissue breakdown, no conclusions could be drawn.

For all the currently described antigens, no sub-analysis was done to determine differences in expression according to clinicopathological characteristics, due to too small sample sizes to perform sound analyses.

### 2.5. Endogenous T Cell Responses to TAA

MAGE-A3, MUC1, Sp17 and hTERT were selected for further assessment of immunogenicity by determining the presence of endogenous TAA-specific T cells after stimulation with overlapping peptides covering the whole TAA. Stimulation with peptide pools covering the viral antigens CMVpp65, EBV BZLF1 and Influenza Matrix Protein 1 were included as a control for assessing immune competence. CD137 up-regulation on CD4^+^ and CD8^+^ T cells after in vitro culture of PBMC with the indicated peptide pools was measured. The clinical characteristics of the patients included in this analysis are shown in Table S6 and the raw data of the T cell responses are shown in Table S7.

**Figure 4:**
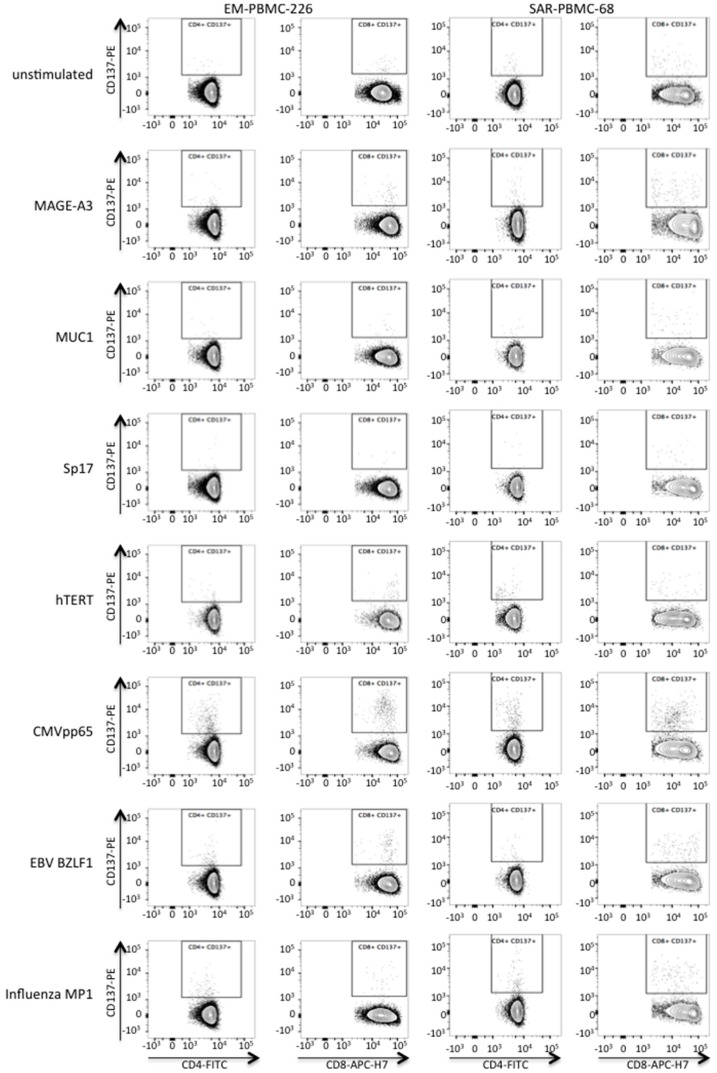
Flow cytometric analysis of antigen-specific T cell responses. For two patients, representative dot plots are shown of CD4^+^CD137^+^ and CD8^+^CD137^+^ T cells after ex vivo stimulation with the indicated overlapping peptide pools. Cells are gated on either CD3^+^CD4^+^CD8^−^CD19^−^ cells or on CD3^+^CD4^−^CD8^+^CD19^−^ cells within the live PBMC gate, as described in the Experimental Section and as depicted in Figure S1.

Virus-specific immunity as determined by CD137 up-regulation on either CD4^+^ or CD8^+^ T cells upon stimulation with at least 1 of the 3 viral antigens was observed in 7/9 (77.8%) of benign controls, 29/31 (93.5%) of EMCAR patients and 14/15 (93.3%) of US patients (data not shown). In Figure 4, we show representative dot plots of the flow cytometric data of two patient samples.

**Figure 5:**
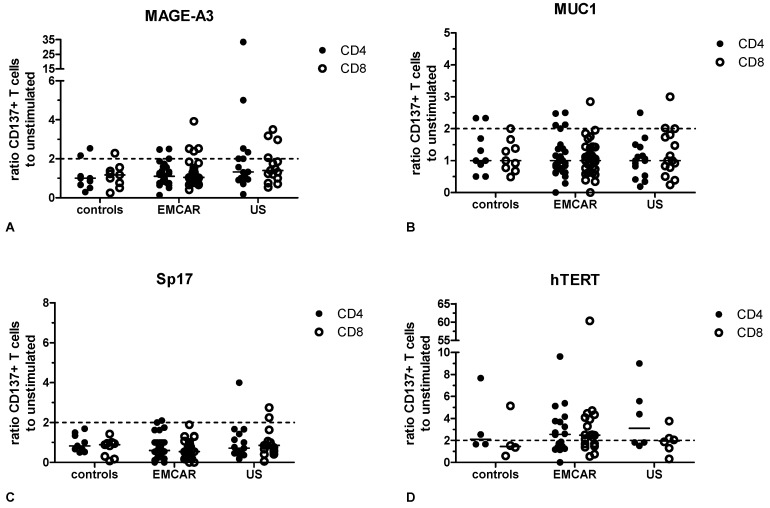
Analysis of T cell responses: (**A**) CD4^+^ and CD8^+^ responses against MAGE-A3; (**B**) CD4^+^ and CD8^+^ responses against MUC1; (**C**) CD4^+^ and CD8^+^ responses against Sp17; and (**D**) CD4^+^ and CD8^+^ responses against hTERT.

MAGE-A3 specific T cells could be measured in 2/9 controls for CD4^+^ T cells and 2/9 controls for CD8^+^ T cells. For EMCAR patients, MAGE-A3 specific CD4^+^ and CD8^+^ T cells were observed in 3/31 and 4/31 patients, respectively, while for US patients MAGE-A3 reactivity could be noted in 6/15 patients for CD4^+^ T cells and in 4/15 patients for CD8^+^ T cells (Figure 5A).

MUC1 specific CD4^+^ and CD8^+^ T cells were present in 2/9 controls for CD4^+^ T cells and in 1/9 controls for CD8^+^ T cells. For EMCAR patients, MUC1 responsiveness could be observed in CD4^+^ T cells in 5/31 patients and in CD8^+^ T cells in 1/31 patients. In US, MUC1 specificity was measured in 1/15 patients for CD4^+^ T cells and in 3/15 patients for CD8^+^ T cells (Figure 5B).

For Sp17, control patients showed no reactivity for either CD4^+^ or CD8^+^ T cells. In EMCAR, Sp17 specificity was seen in 2/31 patients for CD4^+^ T cells, while Sp17 specific T cells could be noted in 1/15 and 2/15 US patients for CD4^+^ and CD8^+^ T cells, respectively (Figure 5C).

hTERT specific T cells were detected in 2/4 and 1/4 control patients for CD4^+^ and CD8^+^ T cells, respectively. In EMCAR patients, hTERT specific T cells were measured in 9/16 patients for CD4^+^ T cells and in 10/16 patients for CD8^+^ T cells. Finally, hTERT reactivity was noted in 3/6 US patients for both CD4^+^ and CD8^+^ T cells (Figure 5D).

Altogether, endogenous T cells specific to any of the tested TAA were only detected in a minority of patients, except towards hTERT where we noted T cell responses in almost half of the patients tested. When looking at all tested antigens, 44% of control patients and 54% of uterine cancer patients showed a T cell response to any of the tested antigens. Furthermore, 22% of control patients and 30% of uterine cancer patients showed a response towards two or more tumor antigens. For some of the tested patients, we investigated whether levels of TAA specific T cells correlate with TAA expression levels in the tumor but we could not note a correlation for any TAA (data not shown).

## 3. Discussion

We have currently validated the expression of five different antigens in uterine tumors, comprising both endometrial carcinoma and uterine sarcoma. To our knowledge, this is the first study to describe the presence of these antigens in uterine sarcoma, with the exception of BORIS [31]. Since the immunogenicity of an antigen, i.e., the ability to elicit an immune response is a key characteristic in order for the antigen to be eligible as a target for immunotherapy, we also assessed whether uterine cancer patients harbored endogenous TAA-specific T cells in their blood. Spontaneous T cell responses against several TAA, mainly melanoma-associated antigens, have been described in several tumor types, both solid tumors and hematological tumors [32]. To our knowledge, spontaneous T cell responses to any of the tested TAA in this study have not been reported to date in either endometrial carcinoma or uterine sarcoma. Furthermore, most studies assessed the immunogenicity to selected TAA-derived peptides in the context of certain MHC molecules, while we used overlapping 15 amino acid peptides covering the whole TAA to overcome MHC restriction and to measure both CD4^+^ and CD8^+^ T cell responses. For most TAA, only low levels of T cell responses could be detected, but we emphasize that this might be due to the experimental conditions used: TAA-specific T cell responses were tested directly ex vivo, without further in vitro restimulation. Thus, the fact that no TAA-specific T cells could be detected in a certain patient does not preclude that TAA-specific T cell precursors are not present, but they might be below the detection limit in these experimental conditions and could become detectable upon long-term in vitro stimulation.

BORIS is a TAA that has emerged fairly recent and has been investigated in several tumor types [33]. BORIS was described in uterine cancer by Risinger et al. [31], to which our results do not entirely correspond. Using a different primer set, they have shown expression in 77% of EMCAR and no expression in normal endometrium. A recent study concerning the expression of BORIS and its paralog CTCF in EMCAR showed that BORIS RNA was significantly increased in non-endometrioid tumors compared with endometrioid tumors [34]. In addition, BORIS levels increased during cancer progression and was associated with aggressive tumors. Importantly, there was an association between BORIS expression and worse five-year disease-free survival. To our knowledge, BORIS-targeted immunotherapy has not been described in the clinic, although a DC vaccine loaded with a truncated form of BORIS has shown promise in the 4T1 mammary mouse tumor model [35].

MAGE-A3 is a cancer-testis antigen that has extensively been studied, both in preclinical models as in the clinic. In contradiction to previously published results by Chitale et al. [36], who found MAGE-A3 to be present in 20% of the analyzed 130 endometrial cancers, we found MAGE-A3 to be present in 90% of the analyzed tumor biopsies, but overexpressed compared to normal endometrium in only 10% of cases. Chitale et al. performed their analysis based on a TMA applying a threshold for positivity of protein expression in >50% of tumor cells, while we currently analyzed the presence of mRNA encoding for MAGE-A3, which allows for the detection of lower levels of TAA expression. MAGE-A3 specific T cells could only be detected in a limited number of patients, which might be due to the very low frequency of MAGE-A3 specific T cells in circulation that may not be detectable with the method used in our study. Inokuma et al. previously showed low level MAGE-A3 T cell responses in healthy females which were increased in breast cancer patients [37], which corroborates our finding of spontaneous MAGE-A3 specific T cells in patients with benign diseases. Our currently analyzed patient population is however too small to evaluate whether these responses are increased in endometrial carcinoma or uterine sarcoma. MAGE-A3 vaccination has a long history in the clinic, especially in melanoma and NSCLC, but although immune responses are frequently induced, clinical effects have been disappointing and have led to the discontinuation of two major phase III clinical trials by GSK due to lack of efficacy. However, due to its interaction with p53 proteins and its expression in cancer stem cells, MAGE-A3 continues to be pursued as an immunotherapeutic approach [38].

Sp17 is a cancer-testis antigen described to be expressed by several tumor types. The percentage of tumors expressing Sp17 in our study is higher than found by Li et al. [39]. However, Li et al. analyzed Sp17 protein expression, which may explain the discrepancy. It may indicate that, even though present as mRNA, it may possibly not be translated into a functional protein in the majority of cases. Next to its presence in uterine tumors, Sp17 has also been shown in ovarian tumors [40,41]. Sp17 RNA was detected in 83% of ovarian tumors. In addition, Sp17 has been shown to be related to chemoresistance in ovarian cancer. Spontaneous Sp17 specific T cells have, to our knowledge, not been described to date, neither in healthy donors, nor in cancer patients. The group of Chiriva-Internati, however, has shown that it is possible to generate Sp17 specific T cells after four rounds of in vitro stimulations with Sp17-pulsed DC in patients with ovarian cancer and multiple myeloma and in healthy donors [42,43,44]. These data suggest the presence of low levels of Sp17 specific T cells in the circulation of both healthy donors and cancer patients. To our knowledge, Sp17-targeted immunotherapeutic approaches have not been tested yet in the clinic, although DC transduced with an adenovirus encoding Sp17 could induce Sp17-specific T cells in vitro that were capable of killing NSCLC tumor cells [45].

MUC1 has been a long time pursued as an immunological target. Our results shown a higher percentage of MUC1-positive tumors with respect to previously published data [46]. MUC1 has been described to play a role in tissue invasion of tumor cells and the formation of metastases [47,48,49]. In our data, we show that there is an increase in MUC1 expression level when comparing normal endometrial samples to primary carcinoma samples as well as to recurrent EMCAR and metastatic lesions, further supporting these data. In US samples, we found MUC1 expression in 43% of tumors. This is unexpected since MUC1 is an epithelial antigen and sarcomas are of mesenchymal origin. Our current tumor population, however, also contains malignant mixed mesodermal tumors, also known as carcinosarcomas, endometrial stromal sarcomas, and adenosarcomas, which both contain an epithelial component where MUC1 is present. Although the majority of the positive sarcomas are attributable to these two types of tumors, we did find other sarcomatous tumors to be positive for MUC1. The presence of pre-existing MUC1 specific T cell immunity in both healthy donors as well as breast cancer patients has been described by Gückel et al., which corroborates our findings of MUC1 reactive T cells in patients with benign diseases [50]. MUC1 targeted immunotherapy has a long clinical experience and has proven to be safe, with a lack of autoimmune responses. However, clinical efficacy is still lacking but the focus is now shifting to the application of MUC1 immunotherapy in the right circumstances, such as combination with chemoradiotherapy and combination with immune checkpoint inhibitors [51].

Telomerase was chosen as target to evaluate due to its role in tumor cell proliferation and survival. hTERT was found to be present in its RNA form in 100% of analyzed EMCAR samples. These results are comparable to previously published results, showing its expression in 78% [52] and 92% [53] of EMCAR samples. The current mRNA and protein data for hTERT expression do not correspond entirely. Although some of the expression levels we found in normal tissue were similar to previously described expression levels [54], we found high hTERT protein expression in brain, kidney, prostate, pancreas and liver, and a negative result for testis tissue. This is quite unexpected and in contradiction to the results of Hiyama et al., and Kyo et al. [52,54]. In addition, we found hTERT protein to be higher in normal endometrial samples compared to EMCAR and US. This phenomenon cannot not be explained solely by hTERT expression in proliferative endometrium, as we also found clear expression in atrophic endometrium, contradicting previous results [55]. These results led us to suspect that the antibody that was used for the immunohistochemistry is likely not ideal. mRNA data showed that hTERT is overexpressed in 71% of tumors compared with normal endometrium and we found an up-regulation in more advanced tumors, supporting the role of hTERT in immortalization and tumor development. hTERT was expressed both as mRNA transcript and protein in sarcoma samples. In addition, we found a significant down-regulation of hTERT in primary and recurrent US in comparison to normal endometrium. In our patient population, vigorous T cell responses to hTERT could be detected. Endogenous T cell responses towards hTERT have been described by several groups in different tumor types and the reported results are quite heterogeneous with regard to the proportion of patients with detectable immunity and the presence of hTERT specific T cells in healthy donors [56,57,58,59,60,61,62,63]. Most studies used only 1 or a few HLA restricted peptides, which considerably constricts the chance of detecting specific T cells. The studies also differed in whether T cells were tested directly ex vivo or first restimulated with peptides in vitro before assessing hTERT reactivity. However, overall these studies are in agreement with our study in that hTERT specific T cells are present in the T cell repertoire of cancer patients. Furthermore, clinical trials with hTERT-targeting vaccines have demonstrated that specific immune responses are induced by these vaccines without major toxicity [64]. Yet, there has been a report of a study in mice by Ugel et al. [65] that showed the development of autoimmune B cell lymphopenia after adoptive transfer of hTERT specific T cells, which was however a temporary phenomenon. Although clinical trials of immunotherapy with hTERT-based vaccines have demonstrated only modest clinical benefit, an optimized vaccine design, more rational patient selection (based on novel insights on hTERT promoter mutations and genomic rearrangements proximal to hTERT) and combination with immune checkpoint inhibitors might offer novel opportunities for hTERT-targeted vaccination [66].

Keeping in mind the recent emergence of mutation-derived neoantigens as targets for immunotherapy, we believe that any future immunotherapeutic approach should also target these antigens. Our study was initiated before the description of the immunogenicity of neoantigens, but their value has been definitely described in POLE-mutated and MSI endometrial tumors [26]. However, the identification of neoantigens is still a cumbersome, time-consuming and expensive approach. Therefore, we propose to use whole-tumor derived approaches for tumor vaccination in uterine cancer, which would target both shared TAA as well as neoantigens. Whole tumor lysate or amplified whole tumor mRNA could be used to load onto DC or nanoparticles for vaccination [67]. Alternatively, tumor-infiltrating lymphocytes (TIL) isolated from uterine tumors and expanded ex vivo are also expected to contain reactivity to both shared TAA and neoantigens [68,69]. Thus, TIL therapy could also be a promising approach for uterine cancers.

In conclusion, the current data collectively validate the presence of the selected TAA in endometrial cancer and indicate the presence of those antigens in uterine sarcoma. In addition, spontaneous T cell responses could be detected in patient blood, indicating the immunogenicity of these antigens. Based on the combined analysis of TAA expression data and the occurrence of TAA-specific T cells, MUC1 and hTERT show the best profile for future application in immunotherapeutic studies, along with the previously validated survivin [70]. However, due to the emergence of neoantigens for cancer immunotherapy, we suggest whole tumor-targeted approaches as optimal vaccination strategies in uterine cancer.

## 4. Experimental Section

### 4.1. Patient Samples, Cell Lines and Reagents

Biopsy material from uterine cancer patients and healthy controls was collected from the tissue biobank of the gynecologic oncology department at the University Hospital Leuven. Whole blood was collected from uterine cancer patients or patients with benign gynecological diseases through venipuncture using EDTA-coated BD vacutainer tubes (BD Diagnostics, Erembodegem, Belgium); buffy coats of healthy controls, collected in ACD bags, were purchased from the Belgian Red Cross. Freshly collected blood was stored at room temperature and processed within 24 h.

The study was conducted in accordance with the Declaration of Helsinki, and the protocol was approved by the Ethics Committee of University Hospitals Leuven. Informed consent was obtained from all patients donating blood and/or tumor tissue prior to inclusion in the study.

Expression levels in normal tissues were analyzed using RNA isolated from normal tissues, purchased from Ambion (Life Technologies, Gent, Belgium) and/or a tissue microarray (TMA) (Pantomics, Inc., Richmond, CA, USA).

The commercial cell lines SKOV-3, OVCAR-3, RL-95-2, HEC-1 and SK-UT-1 were obtained from ATCC (Manassas, VA, USA).

Peptide pools consisting of 15-mer peptides with an 11 aa overlap were obtained from Miltenyi Biotec (Leiden, The Netherlands, MAGE-A3, hTERT, CMVpp65, EBV BZLF1 and Influenza MP1), JPT Peptide Technologies GmbH (Berlin, Germany, MUC1, VNTR repeat region, custom made) or Thermo Scientific (Gent, Belgium, Sp17, custom made).

### 4.2. RNA Isolation and Reverse Transcription Reaction

Total RNA was isolated from snap-frozen material using Trizol (Life Technologies), according to manufacturer’s instructions. 0.2 µg RNA was reverse-transcribed in a total volume of 20 µL in a mixture containing 10X RT buffer, 5.5 mM MgCl_2_, 10 mM dNTP’s, 2.4 µM random hexamers, 0.5 U/µL RNase inhibitor and 50 U/µL Multiscribe reverse transcriptase (all from Life Technologies). The reaction was performed using ABI prism^®^ 7000 Sequence Detection System (Life Technologies) as follows: 10 min at 25 °C, 30 min at 48 °C and 5 min at 95 °C.

### 4.3. Quantitative Real-Time PCR

Quantitative real-time PCR (qRT-PCR) was performed by using ABI prism^®^ 7000 Sequence Detection System (Life Technologies) and the data were analyzed with 7000 system software. Tumor cDNA was analyzed together with testis cDNA as positive control. qRT-PCR reactions were performed in triplicate for all samples. Thermal cycling conditions were as follows: 2 min at 50 °C, 10 min at 95 °C and 40 cycles of 15 s at 95 °C and 1 min at 60 °C. Primers used for the different genes are indicated in Table 3.

TAA gene expression levels were normalized to the geometric mean of β-actin and β-glucuronidase and the mean normalized expression (MNE) was calculated with Q-Gene software. Samples in which cycle threshold (*C*_t_) values of housekeeping genes (β-actin and β-glucuronidase) were above 30 were considered to be of insufficient quality and omitted from analysis. Samples in which no *C*_t_ value for a gene of interest could be measured were considered undetectable (i.e., no expression). All PCR data were expressed as fold change compared to the mean MNE of normal endometrium samples. TAA were considered to be overexpressed in case of ≥2-fold increase. Statistical analysis to calculate differences in expression levels between groups were performed with GraphPad Prism (La Jolla, CA, USA) software, using the appropriate (non)-parametric test following initial analyses to determine normal distribution of results using the D′Agostino and Pearson omnibus normality test.

### 4.4. Immunohistochemistry

For all stainings, 4.0 µm phormol-fixed, paraffin-embedded tissue sections were stained. Sections were first deparaffinized and rehydrated in ethanol. Next, endogenous peroxidase was blocked in 0.5% (MUC1) or 3% H_2_O_2_ (hTERT) in methanol for 30 min. For hTERT, heat-induced epitope retrieval (HIER) was performed at 85 °C. For MUC1, the same step was performed for 1 h at 90 °C in citrate (pH 6.0), after which the slides were cooled down and primary antibody incubation was performed. Ms α Hu MUC1 (DAKO, Heverlee, Belgium, M0613; 1.15 µg/mL) incubated for 2 h at room temperature (RT). Rb anti Hu TERT (Rockland Inc., Limerick, PA, USA, 600-401-252; 2.5 µg/mL) incubated overnight at 4 °C. For all antigens, EnVision+ system HRP-labelled polymers (DAKO) were used as secondary antibodies and incubated for 30 min at RT. Visualization was performed using 3,3′-Diaminobenzidine (DAB, Sigma-Aldrich, Diegem, Belgium) and hematoxylin counterstaining.

The validation of the tumor associated antigens on normal tissues was done using a tissue microarray (TMA) (MTU951; Pantomics, Inc.), following the same protocols.

### 4.5. Slide Scoring

For evaluation of antigen expression, a scoring system was developed based on 2 different parameters: percentage of tumor cells staining positive for the antigen and the staining intensity. For each parameter, 3 different levels were determined. The percentage of tumor cells staining positive was divided in 1%–25%, 25%–50% and >50%. The staining intensity was evaluated as weak (score 1), moderate (score 2) and strong (score 3). Based on these parameters a total score was assigned to each slide (Table 4). All slides were analyzed for the presence of tumor using a conventionally stained HE slide.

### 4.6. PBMC Isolation

For healthy donors, buffy coats were diluted 1/3 in PBS and PBMC were isolated using Lymphoprep™ (AXIS-SHIELD, Dundee, Scotland) density gradient centrifugation according to manufacturer’s instructions and counted with Türck’s solution (Millipore, Overijse, Belgium). For patient samples, peripheral blood was diluted 1/2 in PBS and isolated following the same protocol. The median PBMC number obtained was 8.5 × 10^6^ cells per 10 mL of whole patient blood. PBMC were cryopreserved in 90% human AB serum (Sera Laboratories International, West Sussex, UK) containing 10% DMSO at (5–10) × 10^6^ cells per vial using CoolCell freezing containers (BioCision, San Rafael, CA, USA). The freezing container was placed at −80 °C and vials were transported to liquid nitrogen after 16–48 h and stored until further use.

### 4.7. Detection of TAA-Specific T Cells

The CD137 assay was carried out in T cell medium (IMDM (Life Technologies) supplemented with 5% human AB serum, penicillin–streptomycin (Life Technologies) and l-glutamin (Life Technologies)) and the serum lot was pretested for assay performance. On Day 1, 1–2 vials of PBMC were thawed in a 37 °C water bath until only small ice crystals were visible. The cell suspension was transferred to ice cold RPMI1640 (Life Technologies) and centrifuged at 1500 rpm for 5 min at 4 °C. The PBMC pellet was re-suspended in T cell medium, containing 10 U/mL DNase1 and left to recover at RT for 1 h. After 1 h, cells were counted with trypan blue and re-suspended in T cell medium at a concentration of 10^7^ cells/mL. Cells were plated at 20–100 µL per well in half-well 96 well plates (Greiner Bio-One, Vilvoorde, Belgium) and cultured in the absence or presence of the indicated overlapping peptide pools at 0.6 nmol/mL for 16–20 h at 37 °C/5% CO_2_. After the incubation period, the cells were used for flow cytometric analysis.

### 4.8. Flow Cytometry Staining and Analysis

Plates were centrifuged during 5 min at 1800 rpm and 4 °C. After aspiration of the supernatant, cells were first washed with cold PBS (Life Technologies), centrifuged again and subsequently incubated for 30 min with 150 µL per well of a fixable viability dye (Fixable Viability Dye eFluor 506, eBioscience, Vienna, Austria) diluted at 1 µL/mL in PBS for discrimination of live and dead cells. After incubation, plates were centrifuged and cells were washed with PBS. Next, Fc receptor blocking was performed by adding 25 µL per well of a 10% normal goat serum (Sigma-Aldrich) solution in PBS/0.5% BSA. To identify antigen-specific T cells, the cells were stained with the following antibodies: CD4-FITC (BD Pharmingen, Erembodegem, Belgium), CD137-PE (BD Pharmingen), CD3-PerCp-Cy5.5 (BioLegend, London, UK), CD19-PE-CY7 (BioLegend) and CD8-APC-H7 (BD Pharmingen). All antibodies were titrated to optimal dilution before use. Acquisition was performed with a FACSCanto II flow cytometer using BD FACSDIVA software v6.1.3 (BD Biosciences, San Jose, CA, USA) and (2.5–5) × 10^5^ cells were acquired per sample. Photo Multiplier Tube (PMT) voltages were set using CS & T beads and compensation was calculated using single-stained samples. Data analysis was done using FACSDIVA v6.1.3 (BD Biosciences).

The following gating strategy was used: first, dead cells were gated out using the viability dye and cells were gated on the lymphocyte population based on FSC/SSC properties. Within the lymphocyte population, a gate was set around CD3^+^ CD19^−^ cells and, subsequently, the CD4^+^ and CD8^+^ population was gated in the CD3^+^ population with exclusion of double-positive T cells. Finally, CD137 expression within the CD4^+^ or CD8^+^ population was evaluated. Background expression of CD137 by the T cells was determined on T cells from cultures without peptides. Positive reactivity to an antigen was predefined as a minimum 2-fold increase in CD137 positivity compared to the unstimulated control. The gating of a representative sample is shown in Figure S1.

These studies were conducted in a laboratory that operates under exploratory research principles using investigative protocols and assays.

### 4.9. Statistical Analyses

All statistical analyses were conducted using GraphPad Prism software. Tests were 2-sided and values were considered significant at *p* ≤ 0.05.

## 5. Conclusions

Concerning the TAA expression at the mRNA level, we showed that *MAGE-A3* and *Sp17* were overexpressed in only a minority of patients, *BORIS* was overexpressed in a moderate proportion of tumors, while *hTERT* was overexpressed in most tumors and its expression was upregulated in FIGO stages II, III, and IV EMCAR patients compared to FIGO stage I.

At the protein level, MUC1 was upregulated in primary, recurrent and metastatic EMCAR tumors and in metastatic US tumors compared to normal tissues. For hTERT, the unexpected expression in some normal tissues and our finding that expression was upregulated in normal endometrium led us to question the usefulness of the antibody.

Vis-à-vis the endogenous T cell responses towards these antigens in patient blood samples, we detected reactivity towards MAGE-A3, MUC1 and Sp17 in only a minority of patients, while hTERT-specific endogenous T cell immunity could be detected in almost half of the patients tested.

Altogether, these results point to MUC1 and hTERT as the most promising targets for application in immunotherapeutic strategies for endometrial carcinoma and uterine sarcoma patients. However, considering the value of neoantigens as immunotherapeutic targets, we propose to use whole tumor-derived approaches for tumor vaccination in uterine cancer.

## Figures and Tables

**Table 1:** Evaluation of TAA expression in patient biopsies by qRT-PCR.

TAA ^1^	Samples	Healthy Controls	Endometrial Carcinoma	Uterine Sarcoma
***BORIS ^2^***	# ^3^ samples tested (% ^4^)	9 (100)	49 (100)	15 (100)
	# evaluable samples (%) ^5^	5 (56)	43 (88)	13 (87)
	# undetectable samples (%) ^6^	2 (40)	21 (50)	1 (8)
	% >2-fold change ^7^ (%)		11 (26)	8 (62)
***MAGE-A3 ^8^***	# samples tested (%)	12 (100)	71 (100)	35 (100)
	# evaluable samples (%) ^5^	10 (83)	70 (99)	34 (97)
	# undetectable samples (%) ^6^	0 (0)	7 (10)	3 (9)
	% >2-fold change ^7^		7 (10)	3 (9)
***Sp17 ^9^***	# samples tested (%)	12 (100)	71 (100)	34 (100)
	# evaluable samples (%) ^5^	10 (83)	70 (99)	34 (100)
	# undetectable samples (%) ^6^	0 (0)	0 (0)	0 (0)
	% >2-fold change ^7^		14 (20)	1 (3)
***hTERT ^10^***	# samples tested (%)	9 (100)	70 (100)	34 (100)
	# evaluable samples (%) ^5^	9 (100)	70 (100)	33 (97)
	# undetectable samples (%) ^6^	6 (67)	0 (0)	26 (79)
	% >2-fold change ^3^		50 (71)	16 (48)

^1^ tumor-associated antigen; ^2^ brother of the regulator of imprinted sites; ^3^ Number; ^4^ Percentage; ^5^ A sample is considered evaluable if the *C*_t_ of the reference genes is below 30; ^6^ A sample is considered undetectable when no *C*_t_ value could be measured (i.e., *C*_t_ > 40); ^7^ Numbers are expressed as >2-fold change compared to the mean expression of normal endometrium; ^8^ melanoma-associated antigen A3; ^9^ Sperm protein 17; ^10^ human telomerase reverse transcriptase.

**Table 2:** Evaluation of TAA expression in patient biopsies by IHC.

Tumor Type	MUC1		hTERT	
	#^1^ Samples	%^2^ Positive	# Samples	% Positive
Normal tissue	17	82.4	14	92.8
Primary carcinoma	48	91.6	30	66.7
Recurrent carcinoma	11	100	10	90
Metastatic carcinoma	10	90	9	66.7
Primary sarcoma	39	33.3	22	50
Recurrent sarcoma	15	26.7	12	41.7
Metastatic sarcoma	13	69.2	13	76.9

^1^ Number of analyzed samples; ^2^ percentage of positive samples; IHC: immunohistochemistry.

**Table 3:** Primers used for qRT-PCR.

TAA	Primers and Probe
*BORIS*	Human *CTCFL* Taqman gene expression assay from Applied Biosystems (Life Technologies)
*MAGE-A3*	Sense primer, 5′-GTCGTCGGAAATTGGCAGTAT-3′
	Antisense primer, 5′-GCAGGTGGCAAAGATGTACAA-3′
	Probe, 5′-6FAM-AAAGCTTCCAGTTCCTT-MGB-3′
*Sp17*	Human *SPA17* Taqman gene expression assay from Applied Biosystems (Life Technologies)
*hTERT*	Human *TERT* Taqman gene expression assay from Applied Biosystems (Life Technologies)
β-actin	Human *ACTB* (beta actin) Endogenous Control from Applied Biosystems (Life Technologies)
β-glucuronidase	Human *GUSB* (beta glucuronidase) Endogenous Control from Applied Biosystems (Life Technologies)

**Table 4:** Scoring system for evaluation of TAA expression by IHC.

Percent Positive Tumor Cells	Staining Intensity	Assigned Score
1–25	1	0
1–25	2	0
1–25	3	0
25–50	1	0
25–50	2	1+
25–50	3	2+
>50	1	0
>50	2	3+
>50	3	4+

## Supplementary Materials

Supplementary materials can be found at www.mdpi.com/1422-0067/17/9/1525/s1.
