# Supplementary material for: In Vitro Assessment of the Expression and T Cell Immunogenicity of the Tumor-Associated Antigens BORIS, MUC1, hTERT, MAGE-A3 and Sp17 in Uterine Cancer
Source: Int J Mol Sci. 2016 Sep 9;17(9):1525. doi: 10.3390/ijms17091525 (PMC5037800; doi:10.3390/ijms17091525)
Supplement: Supplementary file 1 [file ijms-17-01525-s001.pdf]

Supplementary Materials: In Vitro Assessment of the Expression and T Cell Immunogenicity of the Tumor-Associated Antigens BORIS, MUC1, hTERT, MAGE-A3 and Sp17 in Uterine Cancer

Anke Vanderstraeten, Sandra Tuyaerts, Tina Everaert, Rieta Van Bree, Godelieve Verbist, Cathérine Luyten and Frederic Amant

Table S1. TAA expression in normal tissues and commercial cell lines. (**A**) A panel of normal human tissues was assayed for expression of *BORIS*, *MAGE-A3*, *Sp17* and *hTERT* by quantitative RT-PCR. Values represent the mean normalized expression of the TAA normalized to β-actin and β-glucuronidase; (**B**) A set of commercially available gynecological tumor cell lines was analyzed for expression of *BORIS*, *MAGE-A3*, *Sp17* and *hTERT* by quantitative RT-PCR. Values represent the mean normalized expression of the TAA normalized to β-actin and β-glucuronidase. Cells are coded using a color scale from green (low expression) over yellow and orange to red (high expression).

**(A)**	**Cancer-Testis Antigens**						**Universal Antigen**	
	BORIS		*MAGE-A3*		*Sp17*		*hTERT*	
	MNE	Level	MNE	Level	MNE	Level	MNE	Level
ADIPOSE	1.86E-04	++	8.97084E-07	−	1.48E-03	+++	5.65E-06	+/−
BLADDER	4.10E-04	++	1.83174E-06	+/−	4.50E-03	+++	6.05E-06	+/−
BRAIN	5.44E-04	++	9.74884E-07	−	3.55E-02	++++	4.02E-05	+
CERVIX	1.34E-04	++	1.03168E-06	+/−	7.05E-03	+++	4.29E-06	+/−
COLON	5.50E-03	+++	1.51983E-06	+/−	3.44E-03	+++	1.05E-04	++
ESOPHAGUS	6.22E-04	++	1.25487E-06	+/−	7.77E-03	+++	4.33E-05	+
HEART	6.93E-04	++	2.59929E-06	+/−	1.33E-02	++++	9.93E-06	+/−
KIDNEY	3.16E-04	++	5.30431E-07	−	1.79E-02	++++	2.92E-06	+/−
LIVER	1.82E-02	++++	9.12201E-07	−	2.88E-03	+++	4.35E-06	+/−
LUNG	2.52E-03	+++	1.30764E-06	+/−	1.59E-02	++++	4.93E-05	+
OVARY	1.22E-04	++	6.97133E-07	−	1.27E-02	++++	3.55E-06	+/−
PLACENTA	3.48E-03	+++	8.06421E-06	+/−	2.68E-03	+++	3.27E-06	+/−
PROSTATE	8.90E-04	++	1.99893E-06	+/−	8.18E-03	+++	6.58E-06	+/−
SKEL.MUSCLE	2.77E-03	+++	5.98591E-06	+/−	9.65E-03	+++	2.04E-05	+
SMALL INTEST.	6.56E-03	+++	1.16568E-06	+/−	5.12E-03	+++	1.41E-04	++
SPLEEN	3.21E-03	+++	8.92141E-07	−	3.40E-03	+++	1.00E-04	++

Table S1. *Cont.*

(A)	Cancer-Testis Antigens						Universal Antigen	
	BORIS		*MAGE-A3*		*Sp17*		*hTERT*	
	MNE	Level	MNE	Level	MNE	Level	MNE	Level
TESTIS	3.49E-01	+++++	3.41E-04	++	2.07E-01	+++++	3.22E-04	++
THYMUS	1.18E-04	++	8.32172E-07	−	5.74E-03	+++	1.69E-03	+++
THYROID	1.11E-03	+++	1.32684E-06	+/−	3.75E-02	++++	4.89E-06	+/−
TRACHEA	2.30E-04	++	1.17E-04	++	7.64E-02	++++	3.79E-05	+
UTERUS	1.60E-03	+++	2.36128E-06	+/−	9.01E-03	+++	6.30E-06	+/−
**(B)**	**Cancer-Testis Antigens**						**Universal Antigen**	
	BORIS		*MAGE-A3*		*Sp17*		*hTERT*	
	MNE	Level	MNE	Level	MNE	Level	MNE	Level
SKOV-3	6.90E-04	++	1.49E-05	+	3.03E-02	++++	5.66E-04	++
OVCAR-3	7.45E-03	+++	8.98E-06	+/−	2.50E-02	++++	1.82E+00	++++++
RL-95-2	3.72E-04	++	3.56E-05	+	1.40E-02	++++	1.92E-03	+++
HEC-1	2.04E-03	+++	3.33E-05	+	2.68E-02	++++	4.90E-04	++
SK-UT-1	ND	ND	5.23E-04	++	6.28E-02	++++	2.97E-04	++

ND: not determined.

The mean normalized expression levels are also graded as follows:

MNE	Level
10^{-8}–10^{-7}	−
10^{-7}–10^{-6}	+/−
10^{-6}–10^{-5}	+
10^{-5}–10^{-4}	++
10^{-4}–10^{-3}	+++
10^{-3}–10^{-2}	++++
10^{-2}–10^{-1}	+++++
10^{-1}–10^{0}	++++++

Table S2. Clinical characteristics of tumors used for qRT-PCR analysis. This table shows the clinical characteristics of the patients from whom biopsies were used in qRT-PCR experiments.

**Tumor Type**	**EMCAR**			**US**		
**Tumor-Associated Antigen**	**BORIS**	**Sp17/MAGE-A3**	**hTERT**	**BORIS**	**Sp17/hTERT**	**MAGE-A3**
Characteristics						
Total number of selected patients	49	71	70	15	34	35
**FIGO stage EMCAR(number of tumors)**						
I	24	34	35			
II	8	11	10			
III	11	14	14			
IV	6	11	11			
**Histological grade EMCAR**						
1	17	24	24			
2	5	14	14			
3	14	22	22			
Unknown	13	11	10			
**Histological subtype(number of tumors)**						
Endometrioid	34	48	48			
Serous	8	12	11			
Clear cell	3	3	3			
Mixed	4	8	8			
Carcinosarcoma				6	11	11
Leiomyosarcoma				4	11	11
Rhabdomyosarcoma				1	2	2
Endometrial stromal sarcoma				4	7	8
Adenosarcoma				0	1	1
Mixed				0	1	1
Unknown				0	1	1

Table S3. Protein expression in normal tissue. A tissue microarray containing 22 different normal tissues was analyzed for protein expression of MUC1 and hTERT. Values represent scores defined using the same scoring system that was used for the tumor samples, as described in Table 2.

Tissue	MUC1	hTERT	Tissue	MUC1	hTERT
Adrenal gland	0	0	Liver	0	4
Urinary bladder	4	3	Lung	4	0
Breast	0	1	Ovary	NA	NA
Cerebellum	0	1	Pancreas	3	3
Brain	0	3	Prostate	0	3
Esophagus	3	3	Salivary gland	3	3
Stomach	1	0	Skin	1	0
Small intestine	0	3	Testis	0	0
Colon	1	1	Thyroid	0	1
Rectum	0	0	Cervix	0	1
Kidney	4	3	Endometrium	NA	NA

NA: not analyzed.

Table S4. Clinical characteristics samples IHC analysis. This table shows the clinical characteristics of the patients from whom biopsies were used for IHC analysis.

**Tumor Type**	**EMCAR**		**US**	
**Tumor-Associated Antigen**	**MUC1**	**hTERT**	**MUC1**	**hTERT**
Characteristics				
Total number of patients	62	48	60	47
Total number of tumors	70	52	65	52
**Number of patients**				
Primary	40	27	37	24
Recurrence	8	11	10	10
Metastasis	6	7	10	10
Primary + recurrence	4	0	1	1
Primary + metastasis	4	3	1	1
Metastasis+ recurrence	0	0	1	1
**FIGO stage primary EMCAR [1] (number of tumors)**				
I	17	10		
II	6	4		
III	11	7		
IV	14	9		
**Histological grade EMCAR [2] (number of tumors)**				
1	15	7		
2	6	3		
3	39	32		
Unknown	9	9		
**Histological subtype [3] (number of tumors)**				
Endometrioid	29	17		
Serous	24	21		
Clear cell	11	9		
Mixed	3	2		
Carcinosarcoma [4]	2	2	19	18
Leiomyosarcoma			17	13
Rhabdomyosarcoma			9	7
Endometrial stromal sarcoma			15	10
Adenosarcoma			5	4

[1] Only primary tumors included; [2] All tumors included; [3] All tumors included; [4] Only carcinomatous tumor part included in EMCAR evaluation.

Table S5. Scores of MUC1 and hTERT in primary and/or metastatic and/or recurrent tumor biopsies of the same patient.

Patient ID	Tumor Type	MUC1 Score			hTERT Score		
		Primary	Metastatic	Recurrence	Primary	Metastatic	Recurrence
PT1	EMCAR	3		4	0		4
PT2	EMCAR	2	4		0	3	
PT3	EMCAR	0	4	4	3	0	0
PT4	EMCAR	4	4		3	3	
PT5	US	0		0	0		3
PT6	US	0	4		0	3	
PT7	US	4	0		NA	4	
PT8	US	0	4		NA	4	
Median score		1	4	4	0	3	3

Black shaded cells: No Paraffin Block.; NA: not analyzed.

Table S6. Clinical characteristics samples T cell responses. This table displays the clinical characteristics of the patients from whom PBMC were used to assess T cell responses.

Tumor Type	Healthy/Benign	EMCAR	US
Total number of selected patients	9	16	10
Healthy	0	N/A	N/A
Benign	9	N/A	N/A
FIGO stage			
I	N/A	9	2
II	N/A	2	0
III	N/A	1	0
IV	N/A	3	6
Unknown	N/A	1	2
Histological grade			
1	N/A	6	2
2	N/A	2	0
3	N/A	8	5
Unknown	N/A	0	3
Histological subtype			
Endometrioid	N/A	11	N/A
Serous	N/A	3	N/A
Clear cell	N/A	1	N/A
Mixed	N/A	1	N/A
Carcinosarcoma	N/A	N/A	2
Leiomyosarcoma	N/A	N/A	3
Rhabdomyosarcoma	N/A	N/A	1
Endometrial stromal sarcoma	N/A	N/A	2
Other	N/A	N/A	1
Unknown	N/A	N/A	1

N/A: not applicable.

Table S7. Raw data T cell responses. This table displays the ratio of the percentage of CD137-positive T cells after stimulation with the indicated TAA compared to the unstimulated sample, for each PBMC sample tested in the T cell response assay. For samples scored positive (i.e., ratio ≥ 2), the actual percentage of CD137-positive T cells after stimulation with the indicated TAA subtracted by the percentage of CD137-positive T cells in the unstimulated sample is shown.

Patient Code	Tumor Type	MAGE–A3				MUC1				Sp–17				hTERT			
		$CD4^+CD137^+$		$CD8^+CD137^+$		$CD4^+CD137^+$		$CD8^+CD137^+$		$CD4^+CD137^+$		$CD8^+CD137^+$		$CD4^+CD137^+$		$CD8^+CD137^+$	
		[1] Fold Increase	[2] %	Fold Increase	%	Fold Increase	%	Fold Increase	%	Fold Increase	%	Fold Increase	%	Fold Increase	%	Fold Increase	%
HC–PBMC–002	benign	2.5	0.200	2.3	0.090	1.7	–	2.0	0.070	1.7	–	1.4	–	2.5	0.200	5.1	0.290
OV–PBMC–006	benign	0.7	–	0.5	–	1.0	–	1.0	–	0.7	–	0.3	–	1.7	–	0.6	–
SAR–PBMC–045	benign	0.9	–	0.3	–	1.0	–	0.9	–	1.0	–	1.0	–	ND	ND	ND	ND
SAR–PBMC–050	benign	1.0	–	1.4	–	2.3	0.040	1.7	–	0.7	–	1.0	–	ND	ND	ND	ND
SAR–PBMC–053	benign	1.0	–	1.0	–	0.5	–	0.5	–	1.5	–	0.9	–	ND	ND	ND	ND
SAR–PBMC–054	benign	1.1	–	1.2	–	1.3	–	1.4	–	1.3	–	0.8	–	ND	ND	ND	ND
SAR–PBMC–057	benign	2.2	0.070	1.6	–	2.3	0.080	0.8	–	0.8	–	0.9	–	ND	ND	ND	ND
SAR–PBMC–063	benign	0.3	–	1.2	–	0.9	–	1.3	–	0.5	–	0.1	–	1.6	–	1.5	–
SAR–PBMC–069	benign	0.5	–	0.8	–	0.5	–	0.7	–	0.5	–	0.2	–	7.7	0.400	1.4	–
EM–PBMC–107	EMC	0.8	–	0.7	–	0.9	–	1.1	–	0.4	–	0.8	–	ND	ND	ND	ND
EM–PBMC–110	EMC	1.6	–	0.7	–	1.4	–	1.0	–	0.6	–	0.6	–	ND	ND	ND	ND
EM–PBMC–113	EMC	2.5	0.099	2.5	0.084	2.5	0.099	1.2	–	2.1	0.074	1.3	–	ND	ND	ND	ND
EM–PBMC–114	EMC	1.2	–	1.3	–	1.4	–	1.4	–	0.8	–	1.1	–	ND	ND	ND	ND
EM–PBMC–115	EMC	1.7	–	1.3	–	0.8	–	0.7	–	0.2	–	0.2	–	ND	ND	ND	ND
EM–PBMC–119	EMC	1.6	–	1.1	–	1.0	–	1.0	–	0.8	–	1.0	–	ND	ND	ND	ND
EM–PBMC–128	EMC	1.6	–	0.9	–	2.1	0.100	1.4	–	1.0	–	0.5	–	ND	ND	ND	ND
EM–PBMC–134	EMC	0.9	–	1.0	–	1.2	–	0.9	–	0.5	–	0.3	–	ND	ND	ND	ND
EM–PBMC–146	EMC	2.0	0.020	1.4	–	2.5	0.030	0.6	–	2.0	0.020	0.3	–	ND	ND	ND	ND
EM–PBMC–152	EMC	1.1	–	0.8	–	0.6	–	0.6	–	1.6	–	0.9	–	ND	ND	ND	ND
EM–PBMC–160	EMC	1.3	–	0.8	–	1.0	–	0.9	–	0.6	–	0.6	–	ND	ND	ND	ND
EM–PBMC–199	EMC	1.3	–	1.1	–	0.5	–	0.8	–	0.4	–	0.5	–	ND	ND	ND	ND
EM–PBMC–204	EMC	ND	ND	ND	ND	ND	ND	ND	ND	ND	ND	ND	ND	2.5	0.380	3.3	7.480
EM–PBMC–207	EMC	1.0	–	0.7	–	1.0	–	0.4	–	1.0	–	0.5	–	ND	ND	ND	ND
EM–PBMC–208	EMC	1.0	–	0.9	–	0.8	–	1.4	–	0.5	–	0.9	–	ND	ND	ND	ND
EM–PBMC–212	EMC	1.0	–	1.0	–	1.0	–	1.9	–	0.0	–	0.0	–	0.0	–	4.7	0.260
EM–PBMC–213	EMC	0.6	–	2.5	0.350	0.7	–	1.7	–	1.0	–	0.4	–	1.7	–	3.9	0.670
EM–PBMC–214	EMC	1.3	–	3.9	0.380	1.3	–	2.8	0.240	0.3	–	1.3	–	2.7	0.310	4.1	0.400
EM–PBMC–217	EMC	0.6	–	1.0	–	0.0	–	0.0	–	0.0	–	0.0	–	1.1	–	1.5	–
EM–PBMC–219	EMC	0.9	–	0.8	–	0.7	–	0.7	–	0.1	–	0.2	–	3.3	2.070	2.5	1.860
EM–PBMC–220	EMC	ND	ND	ND	ND	ND	ND	ND	ND	ND	ND	ND	ND	2.6	0.160	0.8	–
EM–PBMC–226	EMC	2.5	0.090	1.8	–	1.5	–	0.6	–	1.0	–	0.2	–	4.2	0.190	4.5	0.450
EM–PBMC–227	EMC	0.8	–	1.8	–	0.8	–	0.9	–	0.2	–	0.8	–	3.7	0.430	2.4	0.130
EM–PBMC–228	EMC	0.5	–	0.4	–	0.3	–	0.3	–	0.3	–	0.2	–	1.3	–	0.6	–
EM–PBMC–234	EMC	0.8	–	0.7	–	0.8	–	1.2	–	1.0	–	0.9	–	3.8	0.110	2.1	0.130
EM–PBMC–237	EMC	0.8	–	1.0	–	1.0	–	1.1	–	0.6	–	1.3	–	1.4	–	1.7	–
EM–PBMC–239	EMC	1.1	–	1.4	–	0.9	–	1.4	–	0.5	–	0.5	–	1.2	–	1.7	–
EM–PBMC–242	EMC	1.9	–	1.1	–	2.0	0.080	0.6	–	1.6	–	1.0	–	5.4	0.350	2.5	0.180
EM–PBMC–244	EMC	0.8	–	0.8	–	1.1	–	0.8	–	0.5	–	0.4	–	1.5	–	1.4	–

Table S7. *Cont.*

Patient Code	Tumor Type	MAGE–A3				MUC1				Sp–17				hTERT			
		CD4+CD137+		CD8+CD137+		CD4+CD137+		CD8+CD137+		CD4+CD137+		CD8+CD137+		CD4+CD137+		CD8+CD137+	
		[1] Fold Increase	[2] %	Fold Increase	%	Fold Increase	%	Fold Increase	%	Fold Increase	%	Fold Increase	%	Fold Increase	%	Fold Increase	%
EM–PBMC–246	EMC	1.4	–	1.3	–	1.1	–	1.1	–	0.9	–	0.2	–	1.9	–	4.3	1.170
EM–PBMC–247	EMC	1.1	–	1.5	–	2.1	0.090	2.0	0.220	1.8	–	0.9	–	5.1	0.330	2.5	0.350
EM–PBMC–251	EMC	1.1	–	2.4	0.250	0.6	–	1.1	–	0.5	–	1.9	–	9.6	2.070	60.3	10.680
SAR–PBMC–052	EMC	0.1	–	0.9	–	0.6	–	1.8	–	0.6	–	0.5	–	ND	ND	ND	ND
EM–PBMC–138	US	33.4	6.160	3.2	3.94	0.5	–	0.9	–	0.4	–	0.9	–	ND	ND	ND	ND
EM–PBMC–166	US	1.6	–	0.8	–	1.7	–	2.0	0.080	1.4	–	2.3	0.100	ND	ND	ND	ND
EM–PBMC–225	US	0.7	–	1.0	–	0.3	–	1.5	–	0.5	–	1.6	–	1.5	–	2.2	0.230
SAR–PBMC–022	US	2.0	0.010	1.2	–	1.0	–	1.0	–	1.0	–	0.4	–	ND	ND	ND	ND
SAR–PBMC–025	US	0.9	–	1.5	–	0.9	–	1.7	–	0.2	–	0.4	–	ND	ND	ND	ND
SAR–PBMC–026	US	5.0	0.240	1.9	–	2.5	0.090	1.8	–	1.7	–	0.9	–	ND	ND	ND	ND
SAR–PBMC–032	US	1.3	–	1.4	–	0.2	–	0.2	–	1.7	–	1.1	–	ND	ND	ND	ND
SAR–PBMC–036	US	2.3	0.080	3.5	0.1	1.5	–	3.0	0.080	0.5	–	2.8	0.070	ND	ND	ND	ND
SAR–PBMC–047	US	1.3	–	2.1	0.19	1.2	–	0.8	–	0.5	–	0.6	–	ND	ND	ND	ND
SAR–PBMC–055	US	0.9	–	0.7	–	1.1	–	0.8	–	0.8	–	0.7	–	ND	ND	ND	ND
SAR–PBMC–062	US	1.0	–	1.3	–	1.0	–	0.4	–	4.0	0.03	0.5	–	9.0	0.080	1.3	–
SAR–PBMC–064	US	0.2	–	0.5	–	0.4	–	0.5	–	0.5	–	0.8	–	1.8	–	0.3	–
SAR–PBMC–065	US	1.0	–	1.2	–	1.1	–	1.2	–	1.1	–	0.1	–	4.4	0.083	2.0	0.187
SAR–PBMC–066	US	2.0	0.070	1.8	–	1.4	–	1.0	–	0.7	–	1.0	–	5.6	0.320	3.8	0.220
SAR–PBMC–068	US	2.5	0.260	3.0	0.95	0.8	–	2.0	0.490	0.5	–	0.9	–	1.8	–	1.9	–

[1] Fold increase indicates the ratio of the percentage of CD137-positive T cells after stimulation with the indicated TAA compared to the unstimulated sample; [2] % indicates the actual percentage of CD137-positive T cells after stimulation with the indicated TAA subtracted by the percentage of CD137-positive T cells in the unstimulated sample. ND: not determined.

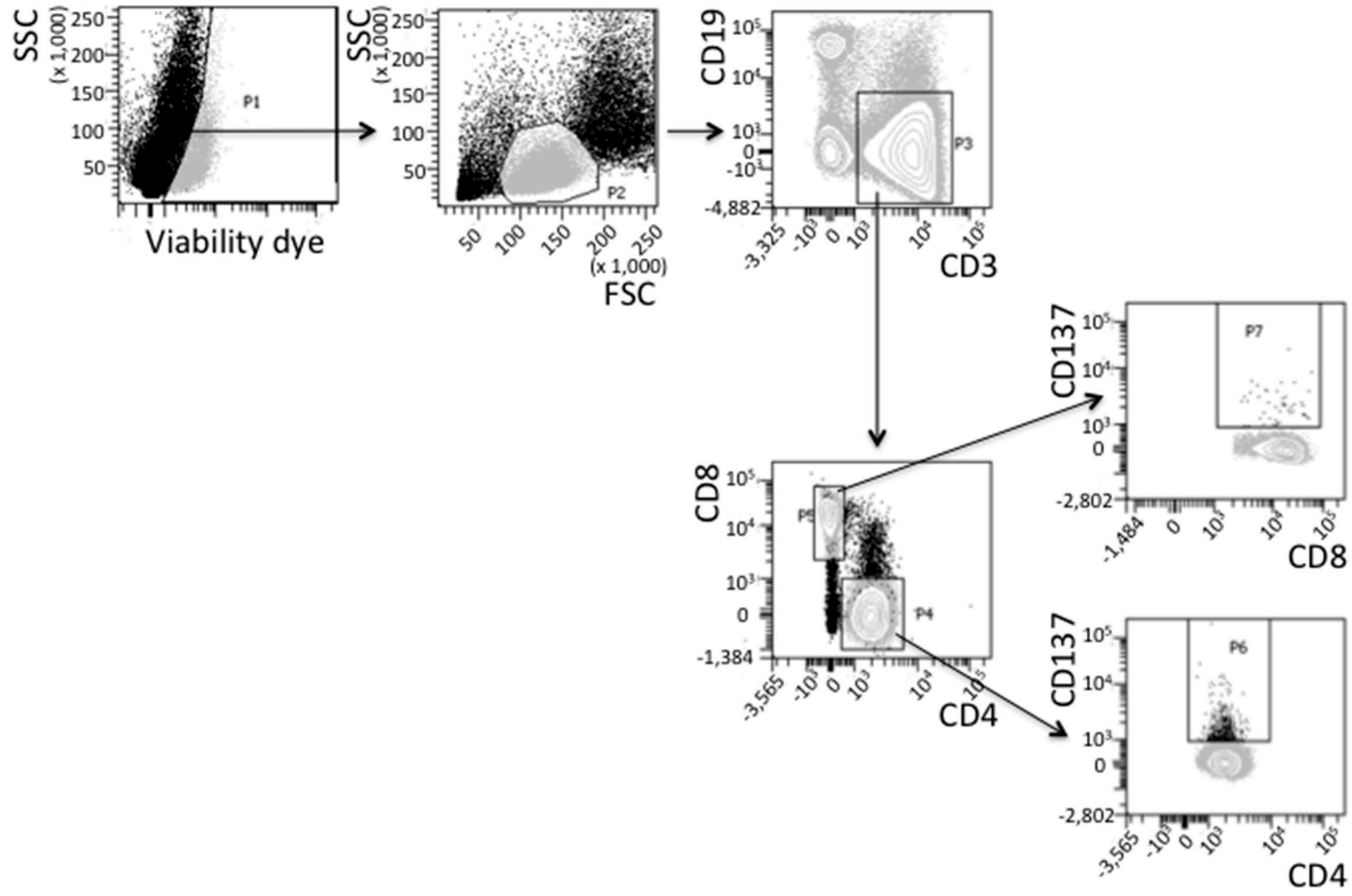

Figure S1. Gating strategy for detection of CD137⁺ T cells in a representative patient sample. This figure shows the gating of a representative sample for analysis of T cell responses.
